# The Critical Shoulder Angle as a Diagnostic Measure for Osteoarthritis and Rotator Cuff Pathology

**DOI:** 10.7759/cureus.11447

**Published:** 2020-11-11

**Authors:** Zak Rose-Reneau, Amanda K Moorefield, Derek Schirmer, Eugene Ismailov, Rob Downing, Barth W Wright

**Affiliations:** 1 Anatomy, Kansas City University of Medicine and Biosciences, Kansas City, USA; 2 Graduate Medical Education, University of Missouri-Kansas City (UMKC), Kansas City, USA

**Keywords:** rotator cuff pathology, osteoarthritis, critical shoulder angle, shoulder, x-rays

## Abstract

The purpose of this study was to correlate critical shoulder angle (CSA), a measurement that takes into account both glenoid tilt and the acromial index (AI), with shoulder pathologies as presented in an earlier study by Moor et al. (2013). Based on Moor et al.’s predicted normal CSA range of 30-35°, we hypothesized that a greater-than-normal CSA would be correlated to or associated with rotator cuff pathology, while a smaller-than-normal CSA would be associated with osteoarthritis (OA). Following Moore et al., we utilized Grashey radiographic imaging because it provides the clearest view of the entire glenoid fossa and acromion. We analyzed 323 anterior-posterior (AP) radiographs to identify and measure the CSA, classifying each patient into one of five groups [none reported (n=94), mild OA (n=156), moderate OA (n=36), severe OA (n=37), and rotator cuff pathology (n=40)]. Our results were statistically significant, supporting the association of smaller CSAs with OA and larger CSAs with rotator cuff pathology. CSA measurements could provide a new means for identifying shoulder pathology and thereby reduce the need for costly and timely imaging techniques. CSA values could also provide useful information to utilize preventatively with interventions such as physical therapy to alter the CSA and reduce the prevalence of OA and shoulder arthroplasties. This study builds on the findings of Moore et al. in creating a correlation between CSA and shoulder pathology.

## Introduction

Even though shoulder pathology remains one of the most common musculoskeletal issues, it often requires multiple diagnostic imaging techniques for proper diagnosis due to the shoulder joint’s structural complexity. Osteoarthritis (OA) and rotator cuff pathology remain two of the most common causes of shoulder pain. We sought to conduct a retrospective study by analyzing 323 anterior-posterior (AP) radiographs, hoping to contribute to the current literature by identifying the correlation between critical shoulder angle (CSA) and shoulder pathology as a preliminary identification method [1]. While previous literature has identified the CSA as useful in identifying OA and rotator cuff pathologies, we aimed to provide clarity on the exact CSAs associated with the different shoulder pathologies. Additionally, we examined the biomechanics of the glenohumeral joint, specifically compressive forces, and how these can create a strain on the supraspinatus muscle.

OA is the most common cause of shoulder arthroplasty, due to mechanically induced injury. The study done by Viehöfer et al. supported this claim by proposing that variation of biomechanics, such as the acromial shape and scapular geometry, could increase compressive forces predisposing individuals to mechanical overloading of the shoulder joint [2]. For example, they found that a higher loading of the supraspinatus muscle is required with larger CSAs, while a shorter acromion requires an increase in deltoid forces, as seen in OA. Furthermore, increased deltoid and supraspinatus forces were necessary for equivalent abduction angles above 45° for the OA group, leading to greater loading of the glenohumeral joint [2]. It was initially proposed by Nyffeler et al. [3] that the acromial index (AI) could be used to understand the development of rotator cuff pathology due to the various acromial shapes either decreasing or increasing one’s susceptibility to rotator cuff pathology. However, the AI was not significant in addressing degenerative changes.

Moor et al. [1] found that degenerative changes of the humerus could actually lead to misleading AI results as the index did not account for the tilt of the glenoid fossa. The significance of using the CSA measurement is that it takes into account both the AI and the glenoid tilt. Furthermore, the accuracy of using the CSA as a means to account for glenoid inclination was confirmed in a study by Daggett et al. [4], in which they concluded that the tilt of the glenoid fossa was linearly correlated with the CSA and demonstrated a significant increase in patients with high/low CSAs and rotator cuff tear pathology. Moor et al. conducted an additional study [5] to follow up on the proposed cause and effect relationship between CSA and glenohumeral joint stability, where they demonstrated that with each increase in CSA, there was a concomitant significant increase in rostral shear forces. CSAs larger than 35° were associated with superior dislocation of the humeral head, demonstrating the impact that glenoid inclination has on CSA and, thus, joint stability.

With this framework in mind, our data was first categorized into normal and abnormal CSAs. The abnormal CSAs were then subcategorized into mild, moderate, and severe cases of OA and CSAs associated with rotator cuff pathology. In establishing parameters for varying levels of joint degradation, including early signs of wear or injury, it allows room for preventive measures to be utilized. Interventions such as physical rehabilitation could be explored as potential means for biomechanical alteration to reduce shoulder joint overload and thereby slow the progression of injury. For example, patients suffering from larger CSAs need assistance altering the consistent strain placed on the supraspinatus muscle, thereby reducing the strain over time and potentially decreasing the rate of supraspinatus injury. Patients with a decreased CSA require alleviation of the compressive forces imposed by the deltoid muscle. The purpose of this study was to identify a correlation between CSA and shoulder pathology as a means for predicting and diagnosing OA and rotator cuff pathologies and thereby decrease the need for expensive and timely diagnostic procedures. We predicted shoulder pathologies based on the CSAs. A CSA less than 30° will be associated with OA and a CSA greater than 35° will be associated with rotator cuff pathology. These associations can then be used as risk factors in a clinical setting for their respective pathological associations.

## Materials and methods

**Figure 1:**
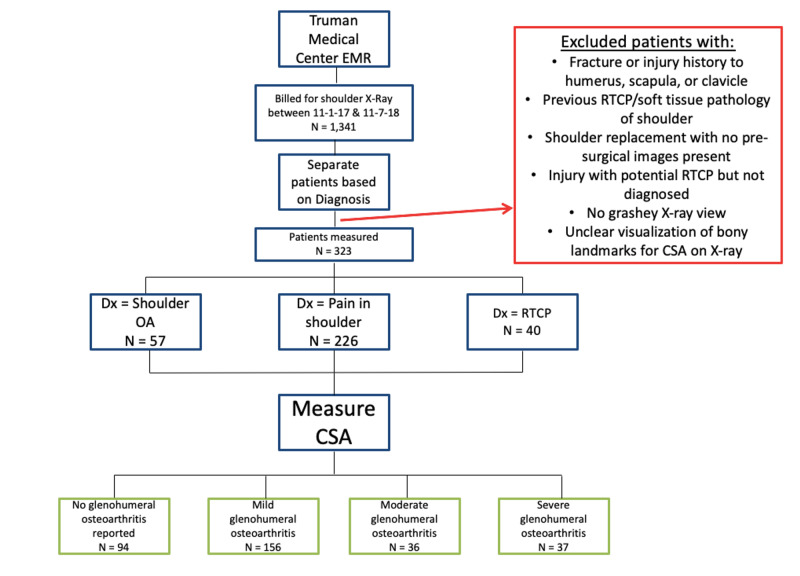
Method of separating groups for statistical analysis EMR: electronic medical record; RTCP: rotator cuff pathology; CSA: critical shoulder angle

This retrospective study included 323 shoulders from 286 individuals (132 women, 154 men) with an average age of 63 years (range: 31-87 years). The sample was selected from patients who had radiographic imaging of their shoulder done between November 1, 2017, and November 7, 2018, at the Truman Medical Center in Kansas City, Missouri (Figure 1). The institutional review board (IRB) approval was granted by the University of Missouri-Kansas City and Truman Medical Center’s IRB boards.

Patients were separated based on their International Classification of Disease, Tenth Revision, Clinical Modification (ICD-10-CM) codes. Patients were excluded if they had a diagnosis of fracture or injury to the humerus, scapula, or clavicle, previous rotator cuff or soft tissue pathology of the shoulder, or shoulder replacement with no pre-surgical images. They were also excluded if Grashey radiographs were not available and/or due to the presence of unclear visualization of bony landmarks for CSA measurement on the radiograph. The final patient cohort included the remaining individuals who were not excluded due to the above-mentioned reasons.

**Figure 2:**
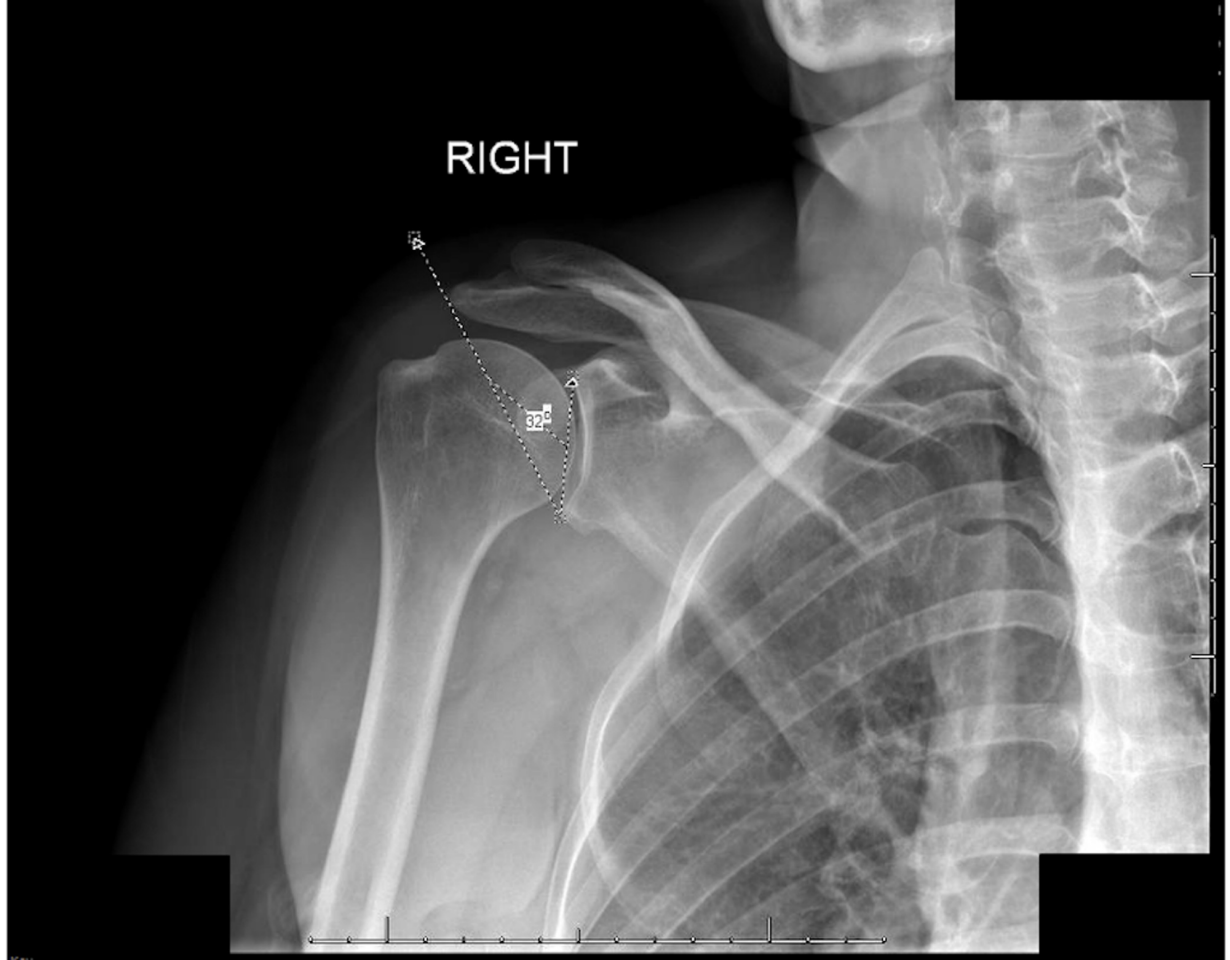
Measurement of the CSA The measurement was obtained by first creating a line from the supraglenoid tubercle to the infraglenoid tubercle, and then creating a line from the infraglenoid tubercle to the lateral most aspect of the acromion CSA: critical shoulder angle

**Figure 3:**
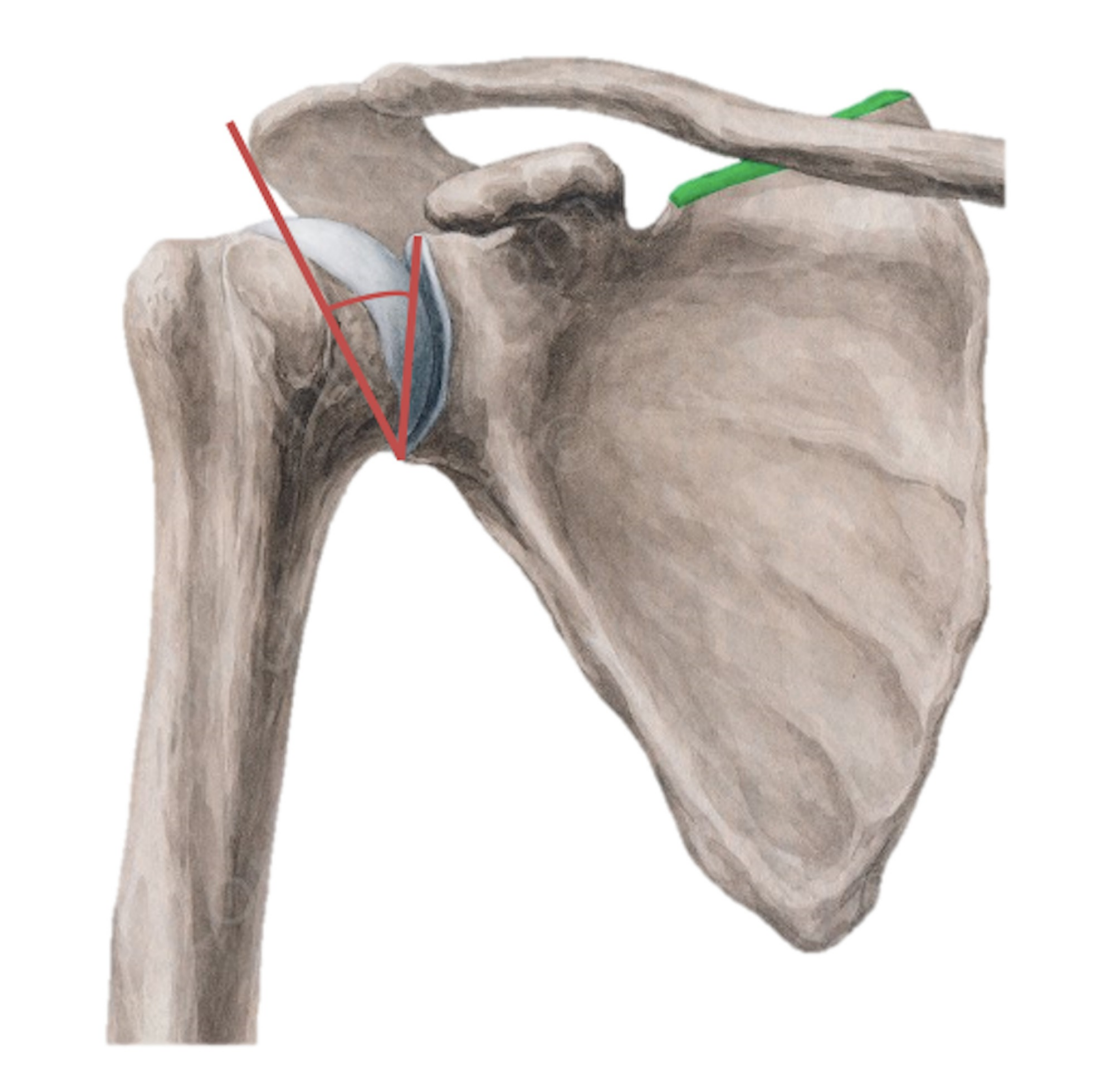
Pictorial description of the critical shoulder angle

Moor et al.’s [1] methodology was used for measuring the CSA to maintain consistency and avoid the possibility of new variables or potential biases. In line with Moor et al., we utilized the Grashey view, which was taken with the patient's back against the imaging receptor, with the patient remaining erect and rotated 30-40° towards the affected side. This specific shoulder view gives the clearest view of the entire glenoid fossa and acromion. Measurements were made using the Cerner Millennium PACS program (Cerner, North Kansas City, MO) to view radiographs. The CSA measurement was obtained by first creating a line from the supraglenoid tubercle to the infraglenoid tubercle, and then creating a line from the infraglenoid tubercle to the lateral most aspect of the acromion (Figures 2, 3) [1]. The angle created between these two lines is the CSA and that angle was recorded. The CSA is a radiological parameter that considers both the AI, which illustrates the lateral extension of the acromion, as well as the tilt (inclination) of the glenoid fossa [1].

The patients were then separated into three groups based on their ICD-10 diagnosis codes [diagnosis of OA (n=57), diagnosis of pain in the shoulder (n=226), diagnosis of rotator cuff pathology (n=40)] and their CSAs were measured. Of note, the CSA measurements in some radiographs were associated with OA despite their lacking an associated ICD-10 OA diagnosis. This explains the greater sample size of OA based solely on CSA measurements compared to the sample size of the ICD-10 OA group. While recording CSA measurements, the radiograph report was read. The report stated the severity of OA seen or the likelihood of a rotator cuff pathology being present. If OA was not reported in the radiograph then “none reported” was recorded. Each patient was then grouped into one of five categories [none reported (n=94), mild OA (n=156), moderate OA (n=36), severe OA (n=37), and rotator cuff pathology (n=40)]. The control group consisted of patients without rotator cuff pathology and no reported OA.

Statistical analysis

Statistical analyses were conducted using Microsoft Excel (Microsoft Corporation, Redmond, WA). An independent samples T-test was performed to compare the five groups. Statistical significance was set at a p-value of <0.05. Mean differences between groups were presented with 95% confidence intervals (CI). Inter-observer reliability was assessed using an independent samples T-test. Spearman's correlation was computed to determine the relationship between the CSA and OA and CSA and rotator cuff pathology. Inter-observer reliability showed minimal bias with a mean difference of 0.55° (SD: 0.58).

## Results

**Figure 4:**
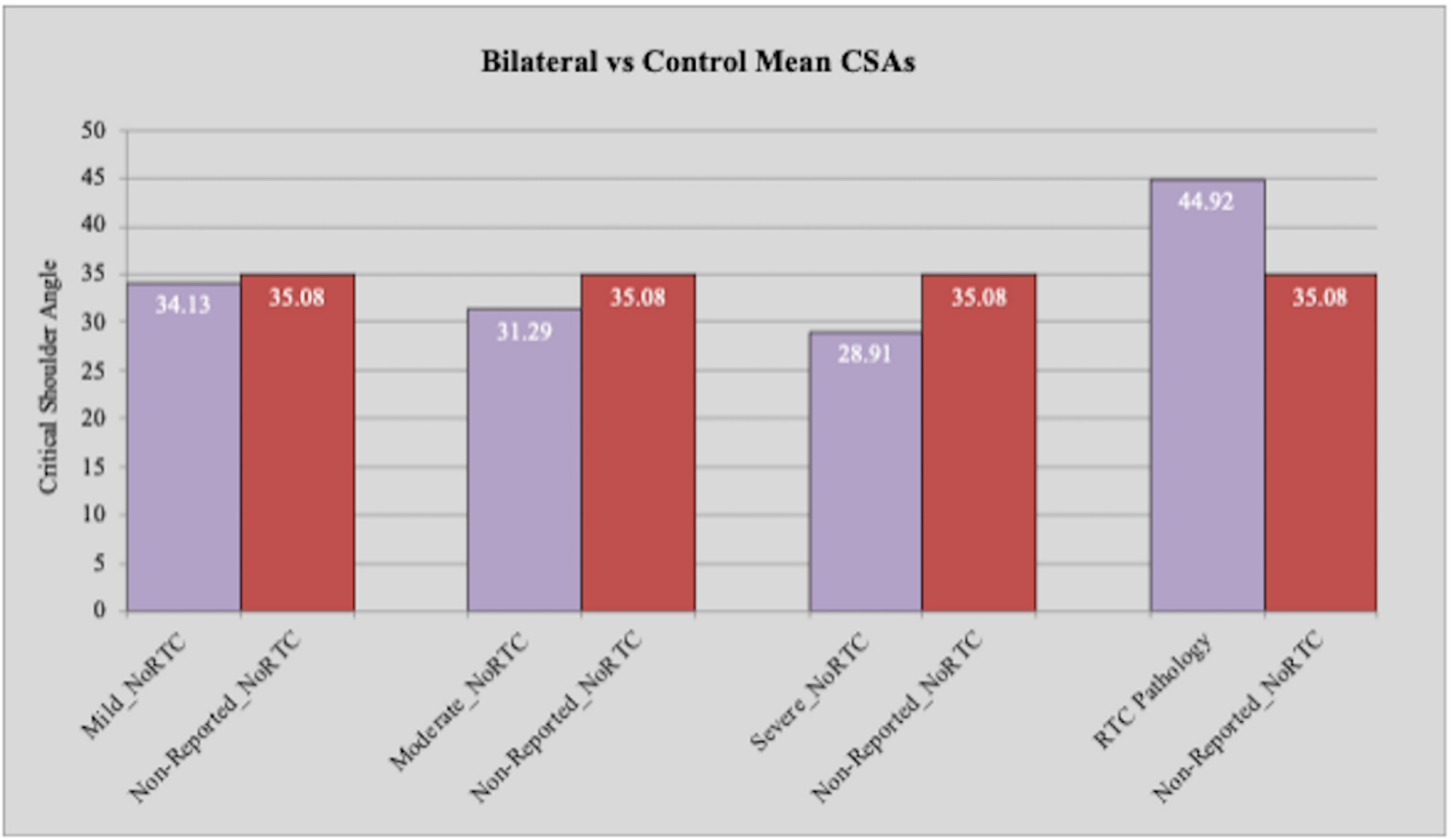
Comparison of critical shoulder angle between bilateral reported OA and rotator cuff pathology and control groups OA: osteoarthritis

Statistically significant differences were found between the means of the control group (patients with no reported OA and no reported rotator cuff pathology) and the moderate OA, severe OA, and rotator cuff pathology groups (p: <0.0001, p: <0.0001, p: <0.0001, respectively) without separation of the right from the left shoulder groups. A mean of 35.08° was calculated for the control group. Means of 31.29°, 28.91°, and 44.92° were calculated for the moderate OA, severe OA, and rotator cuff pathology groups, respectively. The mild OA group had a mean of 34.13°, which was lower than the control group but not significantly different (Table 1, Figure 4).

**Table 1:** Average critical shoulder angle in the different OA and rotator cuff pathology groups combining right and left shoulder angles OA: osteoarthritis

Bilateral					
	None reported = control (no rotator cuff pathology)	Mild OA (no rotator cuff pathology)	Moderate OA (no rotator cuff pathology)	Severe OA (no rotator cuff pathology)	Rotator cuff pathology
N	87	142	34	34	26
Mean	35.08	34.13	31.29	28.91	44.92
Standard deviation	4.00	3.84	3.68	3.93	6.16

**Figure 5:**
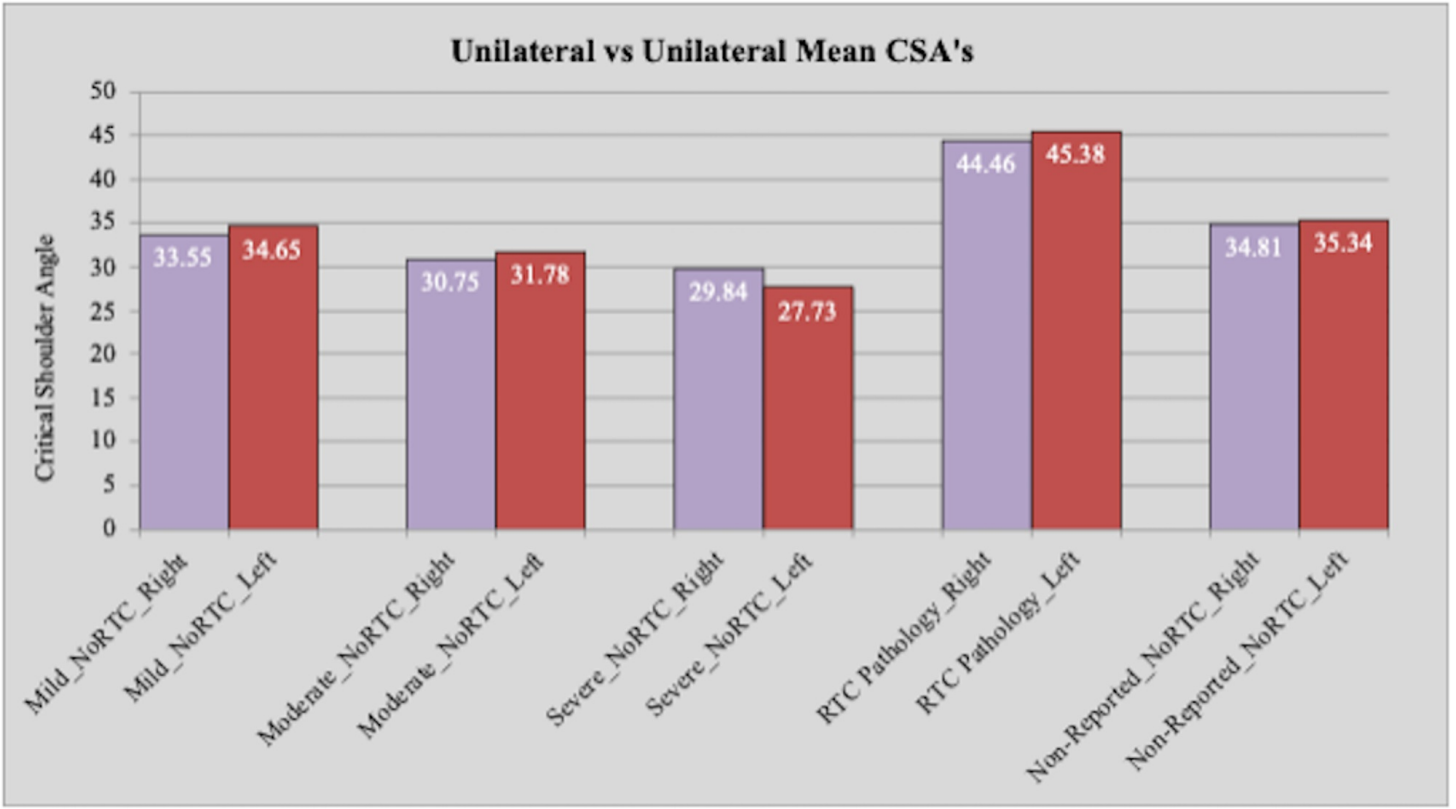
Comparison of critical shoulder angles between right shoulder with reported OA and rotator cuff pathology and left shoulder with reported OA and rotator cuff pathology OA: osteoarthritis

Shoulders were then separated into groups of “right” and “left” and means were compared to eliminate any potential differences caused by shoulder laterality. For the “right” shoulder group, statistically significant differences were found between the control group and the moderate OA, severe OA, and rotator cuff pathology groups (p=0.0007, p: <0.0001, p: <0.0001, respectively). The “left” shoulder group showed consistent results with statistically significant differences found between the control group and the moderate OA, severe OA, and rotator cuff pathology groups (p=0.0022, p: <0.0001, p: <0.0001, respectively). The mean angles for the “right” group were 34.81° for the control groups, 30.75° for the moderate OA group, 29.84° for the severe OA group, and 44.46° for the rotator cuff pathology group. The mean angles for the “left” group were 35.34° for the control group, 31.78° for the moderate OA group, 27.73° for the severe OA group, and 45.38° for the rotator cuff pathology group (Table 2). Means for the “right” and “left” groups were compared within each OA and rotator cuff pathology group and no statistically significant differences were found (Table 2, Figure 5). We found a high correlation between pathology groups and angle measures (p=0.94).

**Table 2:** Average critical shoulder angle in the different OA and rotator cuff pathology groups separating the right and left shoulder angles OA: osteoarthritis

Unilateral										
	None reported = control (no rotator cuff pathology)		Mild OA (no rotator cuff pathology)		Moderate OA (no rotator cuff pathology)		Severe OA (no rotator cuff pathology)		Rotator cuff pathology	
Laterality	Right	Left	Right	Left	Right	Left	Right	Left	Right	Left
N	43	44	67	75	16	18	19	15	13	13
Mean	34.81	35.34	33.55	34.65	30.75	31.78	29.84	27.73	44.46	45.38
Standard deviation	4.00	4.03	3.96	3.67	3.51	3.86	3.80	3.88	6.28	6.27

## Discussion

Moor et al. recorded a mean CSA of 33.1° for the control group, 38° for the rotator cuff pathology group, and 28.1° for the OA group, and concluded that rotator cuff pathology could be predicted at an angle >35° while OA could be predicted by an angle of <30°. Similarly, our results support Moor et al.’s prediction of a normal-range CSA being somewhere between 30-35° [1]. Thus, our data confirmed the significance of CSAs higher than 35° indicating rotator cuff tear pathology and CSAs lower than 30° indicating OA pathology. We also added parameters to include mild, moderate, and severe angles to be consistent with radiology reports. This allows us to not only categorize progression with CSA but also determine if a patient is a good candidate for physical rehabilitation to delay the onset and/or progression of OA. In the case of Moor et al.’s data, a CSA below 30° or above 35° would be abnormal, but in these instances, the pathology is already in the severe category. With a severe CSA, physical therapy would not be as beneficial as a preventative measure as it would be in mild cases.

**Figure 6:**
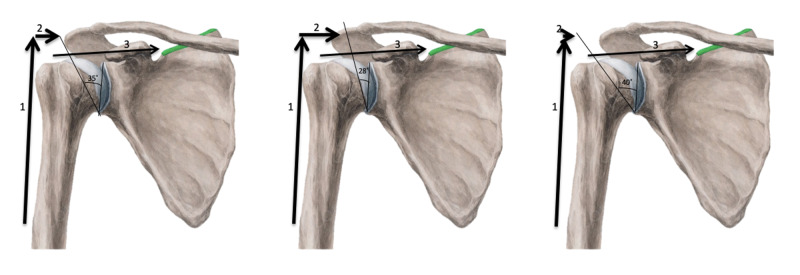
Pictorial description of varying CSA measurements and their impact on compressive forces on the glenohumeral joint Arrow 1: deltoid force vector. Arrow 2: supraspinatus force vector. Arrow 3: compressive force vector of the humeral head on the glenoid fossa CSA: critical shoulder angle

The shoulder joint has extensive mobility and, hence, an increased risk of developing OA associated with joint instability/injury as suggested by Viehöfer et al. [2]. The deltoid muscle has anterior, posterior, and acromial fibers used for flexion/internal rotation of the humerus, extension/external rotation of the humerus, and true humeral abduction, respectively. The rotator cuff muscles and the deltoid have a force-coupling effect to allow for smooth movement of the shoulder joint. An imbalance of these forces can contribute to various types of shoulder dysfunction (Figure 6). In this study, we focus on the supraspinatus muscle specifically as it initiates and regulates the abduction of the humerus and is also the most frequently injured among the four rotator cuff muscles.

More specifically, the supraspinatus helps to centralize the humeral head on the glenoid during deltoid contraction. The articular cartilage arrangement within the glenohumeral joint reduces friction and provides resistance to both shear and compressive forces associated with movement. In OA, a loss of articular cartilage causes excess force and friction. The implications of this are that preventative measures could be taken to reduce compressive forces and decrease a patient’s risk of OA. Preventative measures could include activity modification, gentle range of motion, shoulder stabilization exercises, and isometric strengthening of the rotator cuff musculature. Alternative treatments could also include acupuncture or dry needling and soft tissue massage. Emphasizing such preventative measures could reduce the prevalence of OA/shoulder arthroplasty and rotator cuff pathology. This would also be significant from a financial standpoint as imaging and surgery can be costly and vary immensely in costs.

This is a purely retrospective radiological study utilizing imaging analysis and, hence, we did not obtain any additional patient information such as injury type (i.e., acute vs. chronic), patient scores, demographics, or past medical history. Limitations of this study include a lack of clinical correlation between the patients' radiographs and their symptoms. From an imaging standpoint, some X-rays were clearer than others, which made identifying the CSA challenging. For our data, we provided parameters for mild, moderate, and severe OA, which could vary based on individual subjectivity.

## Conclusions

By utilizing the CSA as a radiological parameter in diagnosing and predicting shoulder pathology, this study created four CSA groups to coincide with our main findings. Groups based on the mean angles included a mild to no OA group between the angles of 33-39°, moderate OA between 30-33°, a <30° CSA group indicative of severe OA, and a ≥40° CSA group indicative of rotator cuff pathology. In establishing clear parameters to identify the progression of shoulder pathology, we could not only utilize preventative measures to delay such progression but also reduce the need for costly diagnostic imaging techniques. Future studies should correlate imaging with clinical presentations including extensive past medical history analysis to see if data is influenced in a significant way and/or should consider measuring pediatric shoulder X-rays as well.

## References

[1] Moor BK, Bouaicha S, Rothenfluh DA, Sukthankar A, Gerber C (2013). Is there an association between the individual anatomy of the scapula and the development of rotator cuff tears or osteoarthritis of the glenohumeral joint?: A radiological study of the critical shoulder angle. Bone Joint J.

[2] Viehöfer AF, Snedeker JG, Baumgartner D, Gerber C (2016). Glenohumeral joint reaction forces increase with critical shoulder angles representative of osteoarthritis-a biomechanical analysis. J Orthop Res.

[3] Nyffeler RW, Werner CM, Sukthankar A, Schmid MR, Gerber C (2006). Association of a large lateral extension of the acromion with rotator cuff tears. J Bone Joint Surg Am.

[4] Daggett M, Werner B, Collin P, Gauci MO, Chaoui J, Walch G (2015). Correlation between glenoid inclination and critical shoulder angle: a radiographic and computed tomography study. J Shoulder Elbow Surg.

[5] Moor BK, Kuster R, Osterhoff G, Baumgartner D, Werner CM, Zumstein MA, Bouaicha S (2016). Inclination-dependent changes of the critical shoulder angle significantly influence superior glenohumeral joint stability. Clin Biomech (Bristol, Avon).

